# The effect of revascularization on mortality and risk of ventricular arrhythmia in patients with ischemic cardiomyopathy

**DOI:** 10.1186/s12872-020-01726-4

**Published:** 2020-10-21

**Authors:** Ahmad Alkharaza, Mousa Al-Harbi, Ihab El-sokkari, Steve Doucette, Ciorsti MacIntyre, Christopher Gray, Amir Abdelwahab, John L. Sapp, Martin Gardner, Ratika Parkash

**Affiliations:** 1 Queen Elizabeth II Health Sciences Center, HI Site, 1796 Summer Street, Room 2501D, Halifax, Nova Scotia Canada; 2 College of Medicine, Imam Abdulrahman Bin Faisal University, Dammam, Saudi Arabia; 3 Research Methods Unit, Nova Scotia Health Authority, Halifax, Nova Scotia Canada

**Keywords:** Implantable cardioverter defibrillator, Coronary revascularization, Coronary artery disease (CAD), And ventricular arrhythmias

## Abstract

**Background:**

There is clear evidence that patients with prior myocardial infarction and a reduced ejection fraction benefit from implantation of a cardioverter-defibrillator (ICD). It is unclear whether this benefit is altered by whether or not revascularization is performed prior to ICD implantation.

**Methods:**

This was a retrospective cohort study following patients who underwent ICD implantation from 2002 to 2014. Patients with ischemic cardiomyopathy and either primary or secondary prevention ICDs were selected for inclusion. Using the electronic medical record, cardiac catheterization data, revascularization status (percutaneous coronary intervention or coronary bypass surgery) were recorded. The outcomes were mortality and ventricular arrhythmia.

**Results:**

There were 606 patients included in the analysis. The mean age was 66.3 ± 10.1 years, 11.9% were women, and the mean LVEF was 30.5 ± 12.0, 58.9% had a primary indication for ICD, 82.0% of the cohort had undergone coronary catheterization prior to ICD implantation. In the overall cohort, there were fewer mortality and ventricular arrhythmia events in patients who had undergone prior revascularization. In patients who had an ICD for secondary prevention, revascularization was associated with a decrease in mortality (HR 0.46, 95% CI (0.24, 0.85) *p* = 0.015), and a trend towards fewer ventricular arrhythmia (HR 0.62, 95% CI (0.38, 1.00) *p* = 0.051). There was no association between death or ventricular arrhythmia with revascularization in patients with primary prevention ICDs.

**Conclusion:**

Revascularization may be beneficial in preventing recurrent ventricular arrhythmia, and should be considered as adjunctive therapy to ICD implantation to improve cardiovascular outcomes.

## Introduction

Sudden cardiac death accounts for at least 300,000 deaths each year in the United States alone [1] with at least 80% occurring in the setting of coronary artery disease [2]. The arrhythmogenic effects of coronary artery occlusion have been well demonstrated in animal and human studies [3–5]. In patients with ischemic cardiomyopathy (ICM), the effect of revascularization on mortality has largely been driven from surgical trials assessing the benefit of coronary artery bypass surgery (CABG) with limited evidence for the mortality benefit of percutaneous coronary intervention in this patient population [6]. In a large randomized trial of CABG versus medical therapy, no mortality benefit was observed as the study was underpowered; however, 10 year follow-up showed overall survival benefit from revascularization [7].. Thus, the overall survival association between coronary revascularization and mortality in this patient population is clear. Nevertheless, the evidence is conflicting regarding the association of revascularization and recurrent ventricular arrhythmia in ICM patients with some trials reporting beneficial association [8, 9] and others reporting no association [10–12]. In this study we assessed the association between revascularization and the clinical outcomes of mortality and recurrent ventricular arrhythmia in patients with ICM and implantable cardiac defibrillator (ICD) utilizing data from a large provincial ICD registry in real world setting.

## Methods

### Study design and patient population

This is a retrospective cohort analysis of patients with ICDs between 2002 and 2014. Data from all patients who underwent ICD or cardiac resynchronization therapy defibrillator (CRT-D) for any reason and had known ischemic heart disease in the province of Nova Scotia, Canada were included. The protocol was approved by the institutional research ethics board. The patient cohort was derived from a comprehensive provincial ICD registry, details of which have been published previously [13]. Patients with inherited cardiomyopathy, ion channelopathies, non-ischemic cardiomyopathy, and those without documented coronary anatomy were excluded from this analysis.

Using the electronic medical record and paper records when required, the following variables were recorded: baseline demographics, clinical characteristics, medications, indication for ICD, cardiac catheterization data, revascularization status (percutaneous coronary intervention, coronary bypass surgery or both interventions), and cardiac non-invasive imaging for CAD including both radionuclide imaging and stress echocardiography. ICD programming was left to the discretion of the electrophysiologist responsible for the care of the patient; shock reduction programming came into effect at the end of this study. The basic programming principles in primary prevention patients were as follows: VT zone range 176–188 bpm, three bursts of ATP followed by the maximum number of shocks, detection intervals varied by device manufacturer but were based on programming in the RAFT study [14]. The VF zone was shock only, with ATP prior to shock delivery in newer generation devices. In secondary prevention patients, the VT zone was set to 20 bpm lower than the documented VT rate, with similar principles to the primary prevention devices for the remainder of the programming. Treatment of ventricular arrhythmias were performed according to current guidelines at the time of this study; this included amiodarone, sotalol, mexilitine or catheter ablation, as indicated [15].

Coronary anatomy and revascularization status were obtained from cardiac catheterization reports and coronary artery bypass graft surgery (CABG) report prior to ICD implantation. If patients had both CABG and percutaneous coronary intervention (PCI), each separate procedure was reviewed to determine the extent of revascularization. Any stenosis greater than or equal to 70% in the territory of the left anterior descending (LAD), left circumflex (LCX) and right coronary artery (RCA) was recorded and deemed significant. Also, any stenosis greater than or equal to 50% in the left main was recorded and deemed significant. Each coronary territory was assessed for status of revascularization and recorded. Based on these findings, patients were divided into two groups based on revascularization status prior to ICD implantation as follows: prior revascularization, and no revascularization groups. Prior revascularization was defined as intervening on all significant coronary artery disease to the greatest extent possible, via PCI, CABG or both.

### Outcome measures

The outcome measures were all-cause mortality, time to first appropriate shock, and time to first appropriate shock or ATP, measured from time ICD implantation. All therapies (shocks and ATP) from the implantable defibrillator were obtained from device interrogation at scheduled or unscheduled clinic visits, remote follow-ups and any emergency department visits or hospitalizations. All devices were capable of continuous monitoring, with storage of any detected events in the device memory. The follow-up schedule was every six months either in-clinic or through the use of remote monitoring, as per current guidelines [16]. All ICD therapies were independently adjudicated for appropriateness by two cardiac electrophysiologists blinded to the cohort allocation of the patient. Any disagreement between the two interpretations was resolved by review with a third electrophysiologist, also blinded. Mortality was obtained from Vital Statistics of Nova Scotia.

#### Statistical analysis

Categorical variables were described as frequencies with percentages; continuous variables were described as mean ± SD. Demographic and clinical characteristics were compared between cohorts using Fisher’s exact test for categorical data and the Student’s t-test for continuous data.

Using Kaplan-Meier analysis, time to either death or ventricular arrhythmia were compared between the no-revascularization group and the prior revascularization group. This analysis was also implemented within each of the Primary and Secondary indication subgroups. The log-rank test was used to test for statistical significance. Time to mortality, and time to ventricular arrhythmia were also assessed individually and effect size data summarized as Hazard Ratios (HR) and 95% confidence intervals (CI) using Cox proportional hazard models. A multivariable Cox proportional hazard model was used to assess the association between revascularization and composite end-point while adjusting for known confounders as well as time to revascularization, identified a priori based on published literature including sex, age, sex, age, ejection fraction, left main or three vessel disease, time to revascularization and creatinine [17, 18]. *P*-values less than 0.05 were considered to be statistically significant. All analyses were performed using SAS 9.4 (the SAS Institute, Cary NC).

## Results

### Population characteristics

**Fig. 1:**
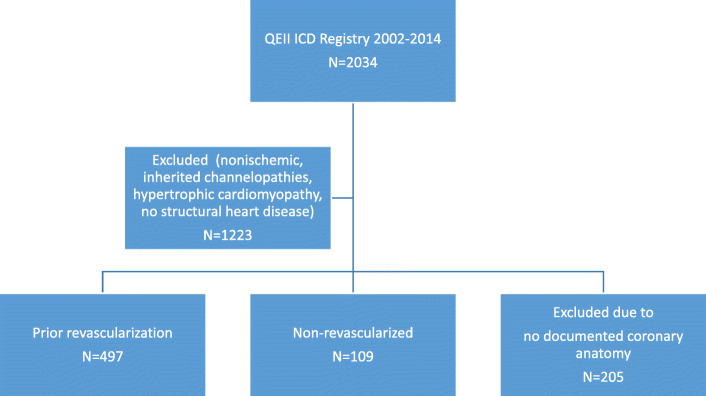
Study flow chart

There were 2034 patients included in the registry from 2002 until 2014. Of these, 606 patients fulfilled the inclusion criteria. Mean follow up was 5.5 ± 3.1 years. Patients were divided into no revascularization (*n* = 109) and prior revascularization (*n* = 497) groups, as shown in Fig. 1. The mean age was 66.3 ± 10.1 years, 72 women (11.9%), mean LVEF for the cohort was 30.5 ± 12.0%. Of the study population 58.9% had a primary indication for ICD and 69.3% had a prior history of heart failure symptoms. Cardiac resynchronization therapy was present in 95 patients (19.1%) in the prior revascularization group, and 19 (17.4%) in the no revascularization group. Coronary catheterization was performed in 89% of the cohort prior to ICD implantation. Revascularization was performed in 95.3% of the cohort prior to ICD implantation, with a median of 3.5 (IQR 0.7,10) years prior to ICD implantation. In the secondary prevention group, this was a median of 2.2 (IQR 0.04, 10) years. The baseline characteristics were similar between the no revascularization and prior revascularization groups (Table 1). Utilizing the Charlson Comorbidity Index, the burden of comorbidity was similar in both groups (5.7 vs 5.6, *p* = 0.45). In addition, baseline medications were similar between groups. Over 90 and 85% of the cohort were treated with beta-blocker and angiotensin-converting enzyme inhibitors/angiotensin receptor blockers at baseline, respectively. (Table 1.)

**Table 1:** Baseline characteristics

Variable	Prior revascularization ***N*** = 497 (%)	No revascularization ***N*** = 109 (%)	***P*** value
**Age**	66.3 ± 10.2	66.4 ± 9.9	0.90
**Women**	61 (12.3)	11 (10.1)	0.62
**Ejection fraction**	31.0 ± 12.2	28.4 ± 11.2	0.04
**Primary Prevention**	292 (58.8)	65 (59.6)	0.91
**Cardiac resynchronization therapy**	95 (19.1)	19 (17.4)	0.82
**Heart Failure**	338 (68.0)	82 (75.2)	0.17
**Atrial fibrillation**	166 (33.4)	38 (34.9)	0.82
**Chronic obstructive pulmonary disease**	82 (16.5)	14 (12.8)	0.39
**Diabetes**	191 (38.4)	34 (31.2)	0.19
**Hypertension**	322 (64.8)	63 (57.8)	0.19
**Hyperlipidemia**	339 (68.2)	63 (57.8)	0.044
**Peripheral vascular disease**	77 (15.5)	16 (14.7)	0.88
**Creatinine (umol/L)**	104.8 ± 61.6	103.2 ± 60.3	0.81
**NYHA Class III**	128/489 (26.2)	32/105 (30.4)	0.29
**NYHA IV**	6/489 (1.2)	3/105 (2.9)	0.95
**Charlson Comorbidity Index**	5.7 (1.7)	5.6 (1.8)	0.45
**Beta Blocker**	460 (92.6)	104 (95.4)	0.40
**Angiotensin converting enzyme inhibitor/angiotensin receptor blocker**	426 (85.7)	93 (85.3)	0.88
**Spironolactone**	98 (19.7)	24 (22.0)	0.60
**Loop Diuretic**	270 (54.3)	60 (55.1)	0.92
**Oral anticoagulant**	159 (32.0)	41 (37.6)	0.26
**Digoxin**	86 (17.3)	24 (22.0)	0.27
**Amiodarone**	88 (17.7)	21 (19.3)	0.68
**Other antiarrhythmic**	16 (3.2)	5 (4.6)	0.56

In this cohort, patients who had received ICD therapy (ATP, shock with ATP or shock without ATP) were grouped based on revascularization status. The median time lapsed from revascularization to ICD therapy was 2.9 years with IQR (0.3–9.8). Among the patients with prior revascularization, 42 patients (8.5%) had appropriate ICD therapy and only 1 patient had received ATP therapy alone. In contrast, among the non-revascularized subgroup, 14 patients (12.8%) had received appropriate ICD therapy, and none received isolated ATP therapy.

### Outcomes

**Fig. 2:**
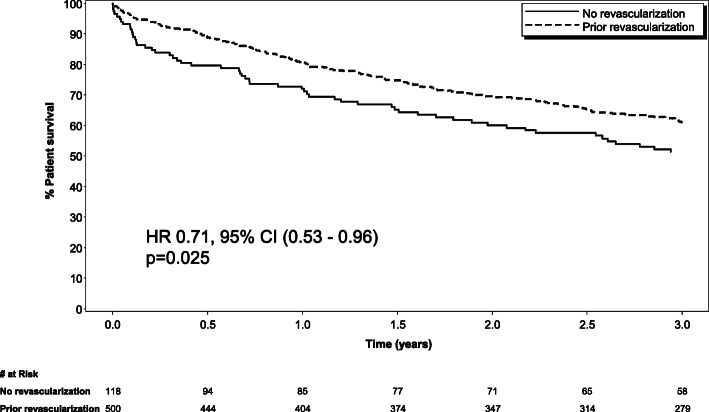
Effect of revascularization on the composite endpoint of mortality and recurrent ventricular arrhythmia. The solid line represents no revascularization; the dotted line represents prior revascularization

**Fig. 3:**
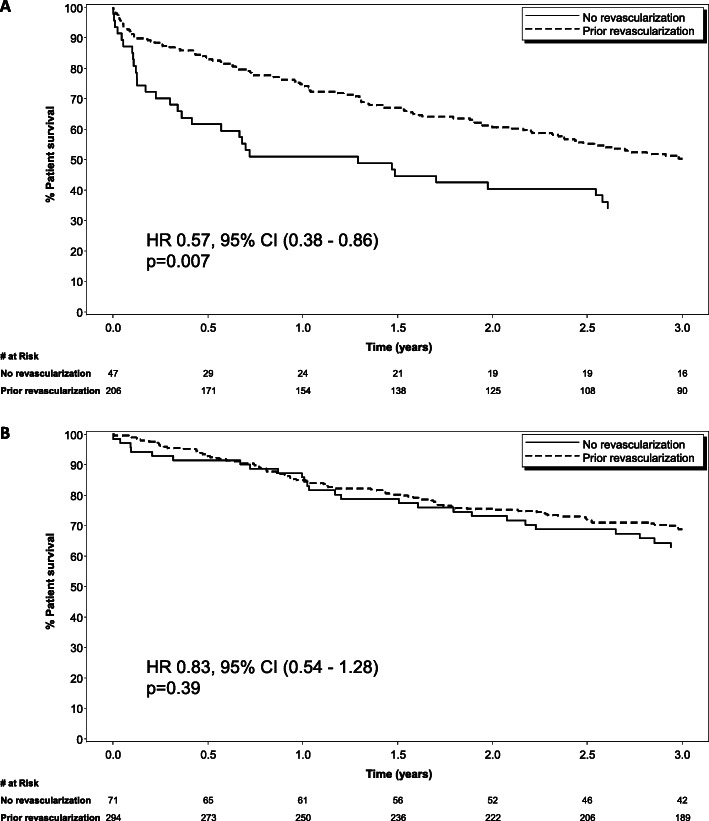
Effect of revascularization on the composite endpoint of mortality and recurrent ventricular arrhythmia by indication for ICD. The solid line represents no revascularization; the dotted line represents prior revascularization. Panel 3A represents secondary indication; Panel 3B represents primary prevention

The extent of coronary artery disease prior to ICD implantation differed between the groups (*p* <  0.001). The revascularized group had mostly three vessel disease (36.6%) while single vessel disease predominated in the no revascularization group (40.4%) (Table 2). In addition, the revascularized group had a higher proportion of patients with significant left main disease when compared to the no revascularization group (18.1 and 2.9% respectively with *p* <  0.0001). (Fig. 2) Among the entire cohort, the total number of deaths during the follow up period was 262 (43.2%). The non-revascularized group had a higher mortality rate (*n* = 61) when compared to the revascularized group (*n* = 201), respectively (60.0% vs 40.4%, *p* = 0.004). Revascularization was associated with a fewer deaths or ventricular arrhythmia events in the overall cohort (HR 0.71, 95% CI (0.53, 0.96) *p* = 0.025). When examining patients by indication for ICD, patients in the revascularization group implanted for secondary prevention had fewer deaths or ventricular arrhythmia events (HR 0.57, 95% CI (0.38,0.86) *p* = 0.007), whereas no statistically significant association was shown on this outcome in patients in the revascularization group with ICDs for a primary prevention indication (HR 0.83, 95% CI (0.54,1.28) *p* = 0.39).(Fig. 3a and b) In analysis of the components of the composite outcome, patients with secondary prevention ICDs who underwent revascularization had lower mortality (HR 0.46, 95% CI (0.24,0.86) *p* = 0.015) and a trend towards fewer ventricular arrhythmia events alone (HR 0.62, 95% CI (0.38, 1.00) *p* = 0.05).

**Table 2:** Extent of coronary artery disease at time of ICD implant

Coronary Disease	Prior revascularization ***N*** = 497 (%)	No revascularization ***N*** = 109 (%)	***P*** value
**Left main stenosis ≥ 50%**	90 (18.1)	3 (2.8)	< 0.0001
**Single vessel disease**	79 (15.9)	44 (40.4)	< 0.0001
**Two vessel disease**	104 (20.9)	24 (22.0)	
**Three vessel disease**	182 (36.6)	14 (12.8)	
**Subcritical disease**	40 (8.0)	17 (15.6)

**Fig. 4:**
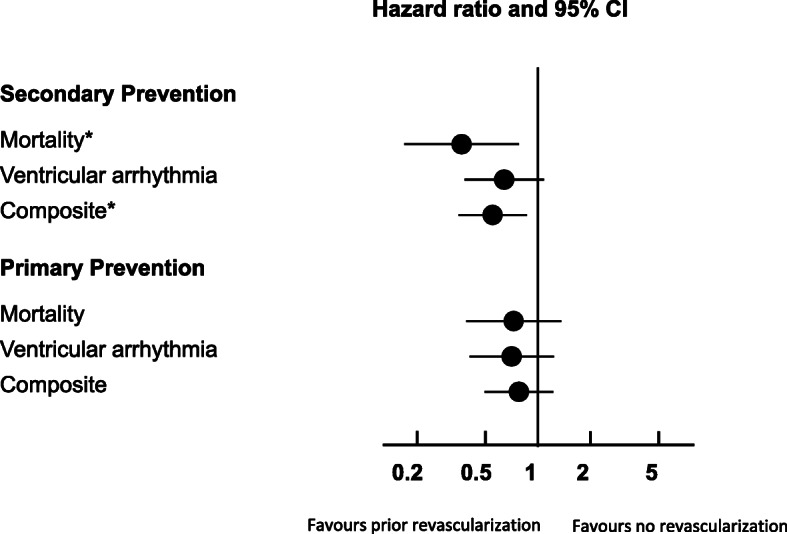
Adjusted Hazard Ratio for mortality, ventricular arrhythmia and the composite outcome in patients with primary and secondary prevention indications for ICD. The variables included in the multivariable model were sex, age, ejection fraction, presence of left main or triple vessel coronary disease and creatinine.(*p < 0.05)

A multivariable analysis was performed after adjusting for the following variables: sex, age, ejection fraction, left main or three vessel disease, time to revascularization and creatinine. Based on this analysis, prior revascularization was associated with significantly fewer composite end-point events in the secondary prevention ICD group (HR 0.35 (95% CI 0.16, 0.77) *p* = 0.009, and HR 0.51 (95% CI 0.32, 0.83) *p* = 0.007, respectively). However, a non-significant trend towards fewer ventricular arrhythmia events was observed in the secondary prevention group (HR 0.59 (95% CI 0.34, 1.03) *p* = 0.064). In addition, time to revascularization in the secondary prevention ICD group did not affect mortality, ventricular arrhythmias or composite end-point. In contrast, the primary prevention ICD group, prior revascularization was associated with fewer ventricular arrhythmias (HR 0.50 (95% CI 0.27, 0.96) *p* = 0.036) but did not affect mortality or composite end point. This is further demonstrated in the forest plot in Fig. 4.

### Types of ventricular arrhythmia and association with ischemia

Of the 316 patients who had appropriate therapy, the type of ventricular arrhythmia was available in 291 patients. The majority of patients had monomorphic VT (*n* = 261, 89.7%), polymorphic VT/VF was present in the remaining (*n* = 30, 11.3%). There was a greater proportion of patients with PMVT/VF with myocardial ischemia (*n* = 14, 70%) compared to patients with MMVT (*n* = 18, 32.7%) (*P* = 0.007). Three patients (15%) with PMVT/VF and two patients (3.6%) with MMVT underwent further revascularization (*P* = 0.15), after receiving ICD therapy. In patients presenting with electrical storm; myocardial ischemia was found in 4/7 (57.1%) patients presenting with PMVT/VF compared to 8/23 (34.8%) patients with MMVT storm. Revascularization was performed in one patient (14.3%) with PMVT/VF storm and one patient (4.3%) with MMVT storm.

## Discussion

In this large cohort study, we found that a history of revascularization was associated with fewer deaths or recurrent ventricular arrhythmia events in patients with a secondary, but not with a primary prevention indication for ICD. This implies that patients with an established arrhythmia substrate might derive more benefit from revascularization. These findings need to be interpreted with caution as our study is at most hypothesis-generating, and does not demonstrate causality. Nevertheless, we did find in multivariable analysis, that these findings remained significant and that revascularization therapy remains an important consideration in patients who have an ICD.

Prior studies have shown conflicting results. In a cohort study of 274 patients with preserved ejection fraction (EF ≥ 40%), revascularization was not associated with a difference in mortality or recurrent ventricular arrhythmia [19]. In the MADIT-CRT trial, revascularization was found to be associated with a significant reduction in mortality and recurrent ICD therapy [9]. In a post-hoc analysis of the MADIT II study, revascularization demonstrated time dependence, with attenuating benefit as time from coronary revascularization increased [20]. In a post-hoc analysis of the VANISH study, patients with more advanced disease that presented with recurrent ventricular arrhythmia despite antiarrhythmic medications. Furthermore, revascularization was not associated with a difference in clinical outcomes [21]. This suggests that the timing of coronary revascularization may have an association with ventricular arrhythmia burden. In addition, our study suggests that this association was more prominent in those patients who have an established arrhythmia substrate, rather than those with a potential substrate.

The beneficial effects of coronary revascularization could be due to restoration of blood flow to hibernating myocardium which may improve cardiac function and limit the progression of ischemic substrate [22]. It is well known that left main disease is associated with a three year mortality of 50% if revascularization is not performed; the optimal form of revascularization has been coronary artery bypass grafting, however, percutaneous coronary intervention has been shown to provide similar outcomes [23, 24]. The association between revascularization and mortality is dependent on the severity of disease and the presence of disease in the left anterior descending (LAD) territory. Prior data supports revascularization to reduce mortality in two and three vessel disease but is equivocal in single vessel unless it is a proximal LAD lesion [25]. In our study, there was a significant proportion of patients with medium to high risk coronary disease who were not revascularized; this was associated with a higher risk of mortality in the overall cohort. From our study and prior data, revascularization has been shown to reduce mortality, and this corroborates to our findings in this population of patients with ischemic heart disease and ICDs. It is also possible that revascularization reduces myocardial ischemia, which is a common trigger for ventricular arrhythmia. This has a wide spectrum, which included not only polymorphic ventricular tachycardia and ventricular fibrillation [26], but also monomorphic ventricular tachycardia [27, 28]. The decision to perform coronary revascularization is often a complex decision that takes into account multiple factors, including the patient’s condition and comorbidities, myocardial ischemia and viability, as well as the technical feasibility of revascularization. These variables could not be considered in the context of our study as is further discussed in our limitations.

There are a number of variables present in this patient population that could have influenced outcomes. Metabolic syndrome, predominant in patients with Type 2 diabetes, has been associated with worse heart failure outcomes in patients with cardiac resynchronization therapy [29], as well as patients with ischemic heart disease [30]. There was no difference in the rate of diabetes in the two groups, and no influence of diabetes on mortality in the multivariable analysis, but the influence of metabolic syndrome could not be further assessed. The effect of inflammatory markers including C reactive protein, brain natriuretic peptide and emerging markers such as heat shock proteins or ST2 could not be measured in this study, but deserve further work to better elucidate outcomes in patients with ICDs.

Remote monitoring (RM) was not used consistently in this study. RM has been shown to be associated with a reduction in mortality and heart failure outcomes [31, 32], but this has not been borne out in randomized clinical trials, with the exception of the IN-TIME Study [33]. RM has demonstrated reduction in the time to detection of arrhythmia and reduction in inappropriate shocks [34]. Further research into determining the effect of RM on this patient population and its outcomes is required to determine effects on total mortality. In this study, all patients were followed every six months for device follow up, there was no loss to follow up in this cohort. The effect of RM use could not be further assessed in this cohort.

There were approximately 1/5 of the population that had cardiac resynchronization therapy (CRT) devices. Response to CRT has been shown to have effects on clinical outcomes including rate of ventricular arrhythmia and overall prognosis [29, 35]. Nevertheless, there is data that demonstrated no difference in left ventricular reverse remodeling irrespective of revascularization [36]. It is also well known that ischemic cardiomyopathy has a lower rate of CRT response, compared to non-ischemic cardiomyopathy [37]. CRT response rates were not available for analysis in this cohort, and may have affect the rate of ventricular arrhythmias, but given the balanced numbers in the two groups and the lack of finding any effect with multivariable analysis, the magnitude of this effect is likely to be small.

### Limitations

There were some limitations to this study. The cause of death was not available for analysis. The study was retrospective in nature, where the decision to perform revascularization was left to physician discretion prior to ICD implantation. The study addressed the revascularization status at the time of the index event, thus there was limited data on revascularization after ICD implantation, with the exception of those with recurrent ICD shocks, as discussed in the results section. The variables considered to perform revascularization were not available for analysis in this dataset, a limitation of the registry. Nevertheless, the concomitant comorbidities that preclude revascularization appear to have an impact on overall mortality, particularly in the secondary prevention group. This finding remains significant and at least provides some indication as to prognosis in this population. Other variables including the effect of metabolic syndrome, heart failure severity, response to cardiac resynchronization therapy, and the use of remote monitoring could not be measured in the groups. Biomarkers such as NT-pro brain natriuretic peptide and C reactive protein, were not in widespread use for prognosis and would have been useful data given its utility in prognostication in this population [30]. An additional limitation is that the power to detect meaningful differences in outcomes in the patients with primary and secondary prevention ICDs as subgroups was limited due to the available sample size in the registry, and overfitting of the Cox models may have occurred. Moreover, myocardial viability was not routinely available in our database for this cohort, but rather a portion of the database; hence, it was not included. The exact cause of death could not be determined, it is possible that death due to cardiac causes was significant in all groups, however, overall mortality remains most important. In addition, since this study spanned 12 years, a temporal effect on mortality and ventricular arrhythmias could be possible but was not evaluated. Finally, selection bias is possible in this study, and causality of ventricular arrhythmia is very unlikely to be related to less complete revascularization alone.

## Conclusion

Our study demonstrated that revascularization was associated with significantly lower mortality events alone, and fewer composite end-point of mortality and ventricular arrhythmias in patients with ischemic cardiomyopathy. This finding was most pronounced in patients with a secondary prevention indication for an ICD, compared to those with a primary prevention indication. Revascularization might be beneficial in patients who present with ventricular arrhythmia, and should be considered as an adjunctive therapy to ICD implantation to improve cardiovascular outcomes.

## Data Availability

The datasets used and/or analysed during the current study are available from the corresponding author on reasonable request.

## Abbreviations

ICD: Implantable Cardioverter defibrillator
LVEF: Left ventricular ejection fraction
EF: Ejection fraction
HR: Hazard ratio
CI: Confidence interval
VT: Ventricular tachycardia
VF: Ventricular fibrillation
CAD: Coronary artery disease
CMP: Cardiomyopathy
ATP: Anti-tachycardia pacing
